# Dietary fat overload reprograms brown fat mitochondria

**DOI:** 10.3389/fphys.2015.00272

**Published:** 2015-09-29

**Authors:** Daniele Lettieri Barbato, Giuseppe Tatulli, Rolando Vegliante, Stefano M. Cannata, Sergio Bernardini, Maria R. Ciriolo, Katia Aquilano

**Affiliations:** ^1^Department of Biology, University of Rome “Tor Vergata,”Rome, Italy; ^2^Università Telematica San Raffaele RomaRome, Italy; ^3^IRCCS San Raffaele RomaRome, Italy

**Keywords:** mitochondria, adipose tissue, nutrition, lipid metabolism, FoxO1, aging

## Abstract

Chronic nutrient overload accelerates the onset of several aging-related diseases reducing life expectancy. Although the mechanisms by which overnutrition affects metabolic processes in many tissues are known, its role on BAT physiology is still unclear. Herein, we investigated the mitochondrial responses in BAT of female mice exposed to high fat diet (HFD) at different steps of life. Although adult mice showed an unchanged mitochondrial amount, both respiration and OxPHOS subunits were strongly affected. Differently, offspring pups exposed to HFD during pregnancy and lactation displayed reduced mitochondrial mass but high oxidative efficiency that, however, resulted in increased bioenergetics state of BAT rather than augmented uncoupling respiration. Interestingly, the metabolic responses triggered by HFD were accompanied by changes in mitochondrial dynamics characterized by decreased content of the fragmentation marker Drp1 both in mothers and offspring pups. HFD-induced inactivation of the FoxO1 transcription factor seemed to be the up-stream modulator of Drp1 levels in brown fat cells. Furthermore, HFD offspring pups weaned with normal diet only partially reverted the mitochondrial dysfunctions caused by HFD. Finally these mice failed in activating the thermogenic program upon cold exposure. Collectively our findings suggest that maternal dietary fat overload irreversibly commits BAT unresponsiveness to physiological stimuli such as cool temperature and this dysfunction in the early stage of life might negatively modulate health and lifespan.

## Introduction

In the modern society the accelerated industrialization has driven a global dietary transition in which traditional dietary patterns are replaced by diets characterized by refined sugars, high content of saturated fats and animal-derived proteins. By 2050 these dietary trends, if unchecked, would be a major environmental risk factor for age-related disorders that shall lower health and life span (Tilman and Clark, 2014).

Dietary saturated fat overload during the intrauterine or postnatal environment affects offspring in a manner that susceptibility to obesity is increased thus leading to metabolic disturbance and development of type 2 diabetes (Chandler-Laney et al., 2012; Khoury et al., 2012). Notwithstanding, the effects of fat overload on adipose tissue physiology of offspring are still underexplored. Notably, brown adipose tissue (BAT) is involved in glucose clearance in the body thus suggesting that its dysfunction during stage of life could participate in age-related metabolic diseases. As consequence, BAT has currently gathered increasing attention as a therapeutic target for overwhelming human obesity and related metabolic disorders (Tseng et al., 2010; Lettieri Barbato et al., 2015).

BAT is an important thermic rheostat by producing heat during cold exposure. Hormonal (e.g., norepinephrine, NE) or pharmacological β_3_-receptor agonists strongly activate the non-shivering thermogenesis in BAT (Goubern et al., 1990; Mund and Frishman, 2013). Under such stimuli BAT mitochondria enhance oxygen consumption and dissipate mitochondrial membrane potential through uncoupling protein 1 (UCP1) in place of synthetizing ATP (Inokuma et al., 2005). OxPHOS complexes integrity is essential for sustaining the gradient proton flux generated by nutrient oxidation, to maintain thermic homeostasis (Cohen and Spiegelman, 2015). Studies in the field of mitochondrial dynamics have identified an intriguing link between BAT activation and mitochondrial rearrangement (Gao and Houtkooper, 2014). Mitochondrial fragmentation is essential in brown adipocytes to assure the high oxidative function of BAT imposed by hormonal inputs governing thermogenesis. However, while hormonal-mediated mitochondrial rearrangement is well-delineated, the impact of nutrients on mitochondria responses of BAT is still unclear. A tissue-specific adaptation to an excess of environmental calories may interfere with mitochondrial dynamics and function in a magnitude that reflects the duration to which the organism was exposed to nutrient overload.

Considerable amount of BAT persists in most adult humans (Saito, 2014). Recently, obesity and diabetes research has put forward mitochondrial “nutrient wasting” in the form of heat as an important concept in metabolic adaptation. This concept is based on the rationale that inducing thermogenesis through increased mitochondrial nutrient oxidation could potentially compensate for the deregulated energetic balance associated with nutrient excess (Schutz et al., 1984; Levine et al., 1999). Consequently, understanding how such mitochondrial “nutrient wasting” process is regulated in BAT might be useful for the treatment of conditions associated with excess of nutrient intake.

The Forkhead box O1 (FoxO1) transcription factor is a unifying regulator of energy metabolism that is abundantly expressed in all tissues and strongly associated with longevity pathways (Kousteni, 2012). While in adipose tissue FoxO1 regulates energy and nutrient homeostasis though energy storage, in brown adipose tissue promotes energy expenditure by enhancing the expression of genes involved in the thermogenic program including peroxisome proliferator-activated receptor γ coactivator (PGC)-1α, and mitochondrial uncoupling proteins (i.e., UCP1,2) and β3-adrenergic receptor (Nakae et al., 2008; Ortega-Molina et al., 2012). Notwithstanding, the way by which nutrients might modulate mitochondrial oxidative metabolism and FoxO1 in BAT is still underexplored.

Herein, we demonstrate that although associated with reduced mitochondrial amount, mitochondrial bioenergetics state was enhanced in BAT of pups exposed to HFD during gestation and lactation. Furthermore, replacement with normal diet is ineffective in completely reverting the inability of adult offspring mice to activate the thermogenic program upon cold exposure. Finally, we show that both in mothers and pups, dietary fat excess reorganizes the network of mitochondria decreasing their fragmentation in a FoxO1-dependent manner.

## Materials and methods

### Mice and treatments

We conducted all mouse experimentations in accordance with accepted standard of human animal care and with the approval by relevant national (Ministry of Health) and local (Institutional Animal Care and Use Committee, Tor Vergata University) committees.

To minimize the number of animals, sample size was calculated by using Sample Size Calculator & Power Analyses Software (http://www.statisticalsolutions.net/pss_calc.php) to reject the null hypothesis of no difference. Eight CD1 adult (2 months-age-old) female mice (purchased from Harlan Laboratories S.r.l., Urbino, Italy) were randomly divided in two groups: normal diet (ND) group (12% kcal from fat, 28% from protein and 60% from carbohydrate, *n* = 4 mice) or high fat diet (HFD) group (60% kcal from fat, 20% from protein, and 20% from carbohydrate, *n* = 4 mice). Dietary treatments were started 8 weeks before mating and then maintained during pregnancy and lactation. Mice were starved overnight (12 h) prior to sacrifice. Litter sizes female mice (*n* = 3 mice each group) were fostered by mothers on the same diet for 4 weeks after birth to yield four groups: pups suckled from ND-fed mothers (ND-f1, *n* = 3 mice) or pups suckled from HFD-fed mothers (HFD-f1, *n* = 3 mice). ND-f1 and HFD-f1 mice were weaned onto the ND at 4 weeks of age. After weaning, the offspring were maintained in ND for 6 weeks (ND-f1-ND and HFD-f1-ND) and exposed to cold (4°C, 3 h; *n* = 3 each group).

All mice were housed with 12 h light/dark cycles and had free food and water access. After cervical dislocation, tissues were explanted and immediately processed.

### Cell lines, treatments, and transfections

3T3-L1 murine adipocytes (American Type Culture Collection, Bethesda, MD, USA) were cultured and differentiated as previously described (Lettieri Barbato et al., 2014a). All experiments were performed in fully differentiated (day 8) 3T3-L1 white adipocytes. T37i murine cell line was kindly provided by Prof. Marc Lombes (Inserm U693, Paris, France) and was grown and differentiated as described by Nakae et al. (2008) with some modifications. Briefly, cells were grown in DMEM/F-12 supplemented with 10% fetal calf serum until confluence. Two days later, the cells were treated with differentiation medium (DMEM containing 10% fetal bovine serum, 0.5 mM 3-isobutyl-1-methylxanthine, 1 μM dexamethasone, 1 μg/mL insulin, 1 μM rosiglitazone, and 2 nM triiodothyronine). The maintenance medium (DMEM supplemented with 10% fetal bovine serum, 1 μM rosiglitazone, and 2 nM triiodothyronine) was changed every 48 h and all experiments were performed after 8 days of differentiation. All experiments were performed in fully differentiated (day 8) T37i brown adipocytes.

Fully differentiated adipocytes were transfected with a siRNA duplex directed against the mouse FoxO1 (Santa Cruz Biotechnologies, Santa Cruz, CA, USA) sequence, plasmid ^cyt−TRAP^FoxO1 (7KQ mutant, gently provided by Prof. Accilli D., Dept. of Medicine, Columbia University, New York, NY) or plasmid HA-FoxO1ADA (^nuc−^FoxO1, Addgene #12143). Transfection with empty vectors or with a scramble siRNA duplex (scr), with no homology to other mouse mRNA, were used as controls. Isoproterenol hydrochloride (Sigma-Aldrich) was dissolved in PBS and added in culture medium at a final concentration of 10 μM and maintained throughout the experiment. Isoproterenol treatment was carried out 30 h after transfection.

### Nuclear, cytoplasmic, and mitochondrial fractioning

Nuclear and cytosolic fractions were obtained using a commercially available NE-PER® extraction kit according to manufacturer's instructions (Thermo Scientific, Milwaukee, WI, USA). Crude mitochondria from BAT were obtained as described by Wieckowski et al. (2009).

### Gel electrophoresis and western blot

Crude mitochondria and BAT were lysed in RIPA buffer (50 mM Tris-HCl, pH 8.0, 150 mM NaCl, 12 mM deoxycholic acid, 0.5% Nonidet P-40, and protease and phosphatase inhibitors). Protein samples were used for SDS-PAGE followed by Western blotting. Nitrocellulose membranes were stained with primary antibodies against α-Actin, TOMM20, GRP75, H2B (Santa Cruz Biotechnologies, Dallas, TX, USA), vDAC, SDHB, UQCRC2, MTCo1 (Abcam, Cambridge, UK), FoxO1 (Cell Signaling Technologies, Danver, MA, USA), Drp1, OPA1 (BD Transduction Laboratories™, San Jose, CA, USA), SDHA, NDUF8 (MitoSciences, Eugene, OR, USA) all diluted 1:1000. Afterward, the membranes were incubated with the appropriate horseradish peroxidase-conjugated secondary antibody, and immunoreactive bands were detected by a Fluorchem Imaging System upon staining with ECL Selected Western Blotting Detection Reagent (GE Healthcare, Pittsburgh, PA, USA). Immunoblots reported in the figures are representative of at least three experiments that gave similar results.

### RT-qPCR analysis and OxPHOS gene transcription ratio

Total RNA was extracted using TRI Reagent® (Sigma-Aldrich). Three micrograms of RNA was used for retro-transcription with M-MLV (Promega, Madison, WI). qPCR was performed in triplicates by using validated qPCR primers (BLAST), Ex TAq qPCR Premix, and the Real-Time PCR LightCycler II (Roche Diagnostics, Indianapolis, IN) as previously described (Lettieri Barbato et al., 2013). mRNA levels were normalized to actin mRNA, and the relative mRNA levels were determined by using the 2^−ΔΔ*Ct*^ method. To calculate OxPHOS gene expression ratio, nuclear-encoded OxPHOS mRNA levels were compared to mitochondrial-encoded OxPHOS mRNA. The relative mRNA levels were determined by using the 2^−ΔΔ*Ct*^ method and were normalized to actin.

### Determination of mtDNA/nDNA ratio

Total DNA was isolated by standard proteinase K digestion, phenol–chloroform extraction, and ethanol precipitation methods. mtDNA copy number was analyzed for each mouse by RT-qPCR according to Yatsuga and Suomalainen (2012), utilizing 12S rRNA gene primers for mtDNA and normalized against the nuclear actin gene.

### Assay of mitochondrial membrane potential

Crude mitochondria were incubated with MitoTracker Red CMX ROS and Mitotracker Green (Life Technologies Ltd.) at concentration of 250 nM (30 min, 37°C) prior to cytofluorimetric analysis through a FACScalibur instrument (Beckton and Dickinson, San Jose`, CA, USA). Mitochondrial population was selected by gating Mitotracker Green positive particles. MitoTracker Red fluorescence was analyzed in 200,000 mitochondria.

### NADH diaphorase staining

Mitochondrial oxidative activity was detected by NADH diaphorase staining using a standard methodology as previously described (Langone et al., 2014). Briefly, the BAT sections were incubated at 37°C for 20 min with staining solution containing nitro tetrazolium blue (NBT) and NADH. The stained samples were fixed for 15 min in 4% formaldehyde and after extensive wash in ddH_2_O were mounted with Mount Quick Aqueous (BIO-OPTICA). The images reported are representative of one experiment out of four that gave similar results.

### Oxygen consumption, respiratory control index, and bioenergetics state

Oxygen consumption rate was determined in minced BAT or crude BAT mitochondria using the Oxygraph Plus oxygen electrode system (Hansatech Instruments Ltd., Norfolk, UK). Real-time oxygen consumption was recorded at 37°C, for 6 min by resuspending BAT or mitochondria in mitochondrial activity buffer (70 mM sucrose, 220 mM mannitol, 2 mM HEPES buffer, 5 mM magnesium chloride, 5 mM potassium phosphate, 1 mM EDTA, 5 mM succinic acid, and 0.1% fatty acid free bovine serum albumin, pH 7.4). Respiratory control index was calculated by the ratio between ADP-activated mitochondrial respiration (state 3) and steady state (state 4) of respiration. Oxygen consumption was normalized for tissue weight (g) or protein concentration. ATP level was detected in total lysate by using ATP Bioluminescence assay kit (Roche Diagnostics) and values were normalized for protein concentration. Bioenergetics state was calculated by normalizing oxygen consumption of BAT for its ATP content.

### Citrate synthase and mitochondrial complex I–III activity

Citrate synthase activity was measured by Citrate Synthase Activity Assay Kit (Abcam, Cambridge, UK). Mitochondrial complex I–III activity was assay as described in Barrientos et al. (2009). All values were normalized for total protein amount.

### Statistical analysis

The results are presented as mean ± S.D. Statistical evaluation was conducted by ANOVA followed by the post Student-Newman–Keuls. Differences were considered to be significant at *P* < 0.05.

## Results

### Dietary fat excess impacts on mitochondrial OxPHOS functionality in adult mice

**Figure 1:**
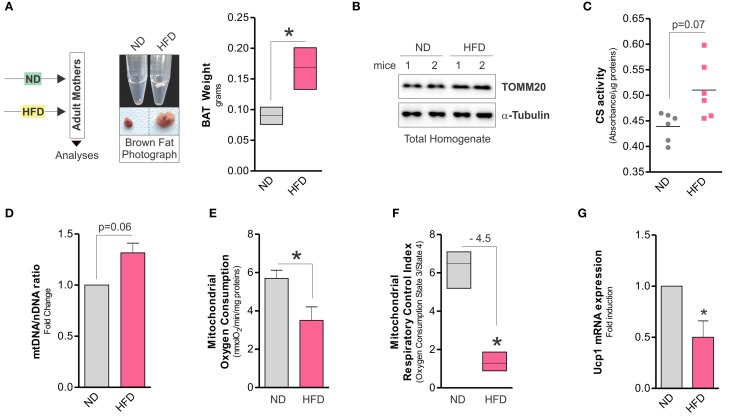
**High fat diet expands BAT mass and affects mitochondrial OxPHOS capacity. (A)** Schematic model of dietary approach in female adult CD1 mice (left) and photograph of BAT aspect (central, bottom), density (central, upper) and weight (right). **(B)** Mitochondrial TOMM20 analyzed by Western blot in total protein lysate of BAT. **(C)** Citrate synthase (CS) activity measured by spectrophotometer in total lysate of BAT. **(D)** Mitochondrial (mtDNA) and nuclear DNA (nDNA) ratio assayed by RT-qPCR. **(E,F)** Real time mitochondrial oxygen consumption **(E)**, mitochondrial respiratory control index **(F)** measured by a polarographic method in crude mitochondria of BAT. **(G)** Ucp1 mRNA expression analyzed by RT-qPCR. Tubulin served as loading control. Bar graphs are expressed as mean ±S.D. (*n* = 4 mice per group; ^*^p < 0.05 vs. ND). HFD, high fat diet; ND, normal diet.

**Figure 2:**
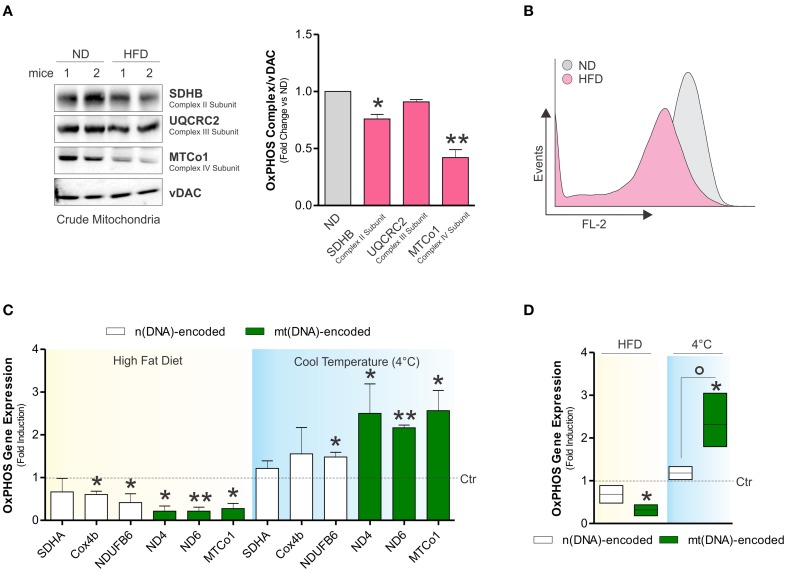
**Dietary fat excess induces alterations of OxPHOS subunits and reduces Drp1 levels in BAT of adult mice. (A)** Mitochondrial OxPHOS subunits analyzed by Western blot in BAT crude mitochondria (left) and relative densitometric analyses (right). **(B)** Mitochondrial membrane potential (ΔψM) detected by cytofluorimetric analysis in BAT crude mitochondria after Mitotracker Red CMXROS staining. **(C)** mt(DNA) and n(DNA)-encoded OxPHOS genes expression evaluated by RT-qPCR after HFD or cold exposure. **(D)** Mean values of mt(DNA) and n(DNA)-encoded OxPHOS genes expression assayed as described in **(C)**. VDAC served as loading control. Bar graphs are expressed as mean ±S.D. (*n* = 4 mice per group; ^*^p < 0.05; ^**^p < 0.01 vs. controls; °*p* < 0.05 vs. n(DNA)-encoded genes). HFD, high fat diet; ND, normal diet.

Although it is clear that dietary regimens rich in saturated fats cause metabolic defects in high oxidative tissues such as skeletal muscle (Corcoran et al., 2007), the impact on BAT function is uncertain. Herein, we observed that HFD increased total body weight (32.7 ± 1.2 vs. 28 ± 4.6 g) and expanded BAT mass in adult mice (Figure 1A), whereas mitochondrial amount was not affected, as demonstrated by Western blot analysis of TOMM20 (Figure 1B), enzymatic assay of citrate synthase (Figure 1C), and qPCR analysis of mtDNA (Figure 1D). However, mitochondrial functionality was profoundly impaired by HFD. In particular, in crude mitochondria we found decreased oxygen consumption (Figure 1E) and respiratory control index (Figure 1F). These events were associated with decreased UCP1 mRNA levels (Figure 1G) and unchanged ATP content (data not shown). Successively, in crude mitochondria of HFD-treated mice we analyzed some OxPHOS protein components and we revealed a diminished amount of both SDHB (complex II subunit) and MTCo1 (complex IV subunit) protein levels without any significant changes in UQCRC2 (complex III subunit) (Figure 2A). Accordingly, we found that HFD caused a reduction of transmembrane potential (ΔψM) (Figure 2B), suggesting that OxPHOS impairment could be responsible for mitochondrial functionality changes imposed by dietary fat excess.

Next, we verified whether HFD-induced decrease of OxPHOS subunits was associated with alteration of their gene expression. As reported in Figures 2C,D, HFD significantly reduced mRNA expression of both mitochondrial (mt)- and nuclear(n)-encoded OxPHOS genes. We then compared the effects of HFD with cold exposure, a validated experimental tool to activate BAT mitochondrial activity (Lim et al., 2012). Oppositely to HFD, the levels of OxPHOS mRNAs were increased in BAT after cold exposure with the (mt)-encoded being the most significantly affected (Figures 2C,D). These results suggest that the imbalance of the expression of OxPHOS subunits could represent a mark of BAT responses to environmental stimuli.

### Maternal dietary fat excess affects mitochondrial mass and enhances bioenergetics state in bat of pups

**Figure 3:**
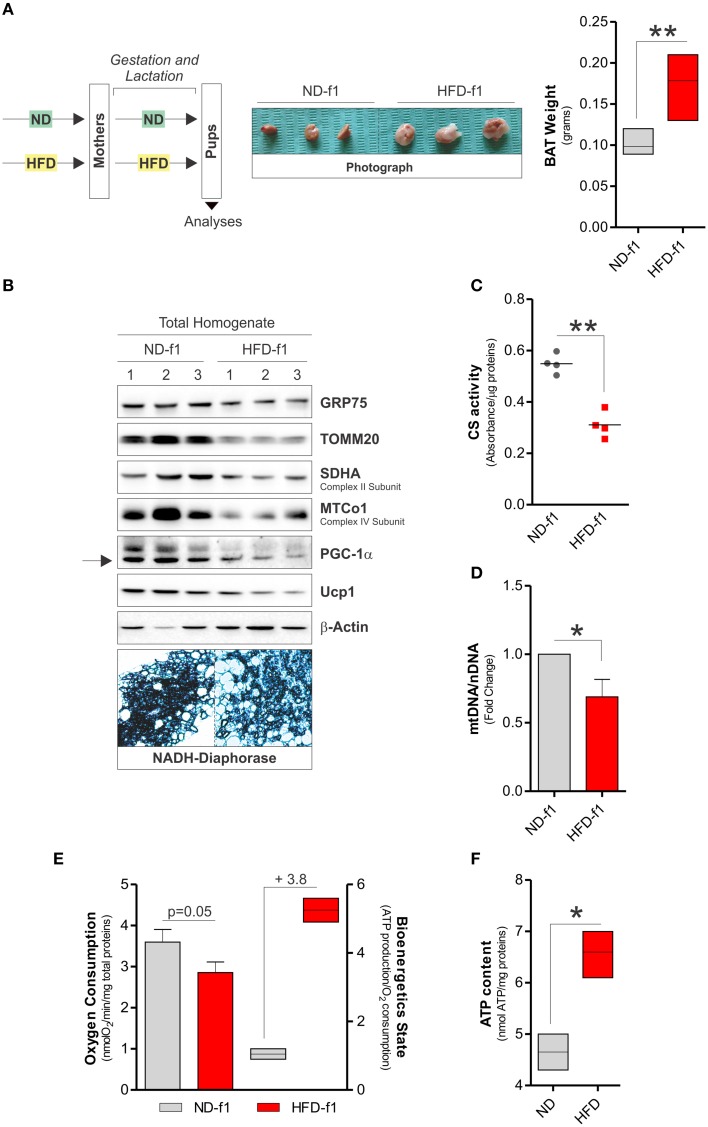
**Fat overload during gestation and lactation induces BAT expansion and mitochondrial mass pauperization. (A)** Schematic model of dietary approach in female pups CD1 mice (left), and photograph of BAT aspect (center), and weight (right). **(B)** GRP75, TOMM20, SDHA, MTCo1, PGC-1α, and Ucp1 proteins analyzed by Western blot in total lysates of BAT. Below are reported BAT sections after NADPH diaphorase staining. **(C)** Citrate synthase (CS) activity measured by spectrophotometer in total BAT lysates. **(D)** Mitochondrial (mtDNA) and nuclear DNA (nDNA) ratio assayed by RT-qPCR. **(E)** Real time oxygen consumption measured by a polarographic method and bioenergetics state expressed as ATP content/O_2_ consumption in total BAT homogenates. **(F)** ATP levels measured by a cheminoluminescent assay in total BAT homogenates. β-Actin served as loading control. Bar graphs are expressed as mean ±S.D. (*n* = 3 mice per group; ^*^p < 0.05; ^**^p < 0.01 vs. ND-f1). HFD-f1, high fat diet offspring; ND-f1, normal diet offspring.

**Figure 4:**
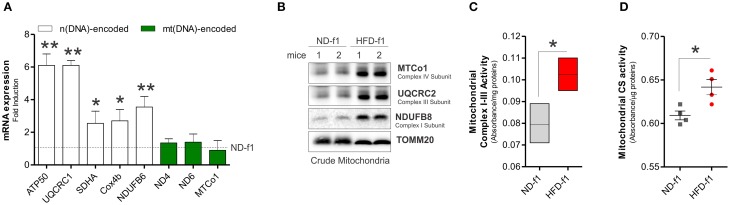
**HFD in the early stage of life alters OxPHOS genes expression and enhances mitochondrial functionality in BAT. (A)** n(DNA) and mt(DNA)-encoded OxPHOS gene expression evaluated by RT-qPCR. **(B)** MTCo1, UQCRC2, and NDUFB8 protein levels analyzed by Western blot in BAT crude mitochondria. **(C,D)** Mitochondrial complex I–III **(C)** and citrate synthase activity **(D)** measured by spectrophotometer in BAT crude mitochondria. TOMM20 served as loading control. Bar graphs are expressed as mean ±S.D. (*n* = 3 mice per group; ^*^p < 0.05; ^**^p < 0.01 vs. ND-f1). HFD-f1, high fat diet first offspring; ND-f1, normal diet first offspring.

We also investigated the mitochondrial adaptations of BAT in offspring mice exposed to HFD during gestation and lactation. In 4 weeks-old offspring grown in HFD (pups, HFD-f1), we observed augmented total body weight (11.6 ± 2.2 vs. 9.5 ± 0.9 g) and higher BAT mass than pups grown in ND (ND-f1) (Figure 3A). We then evaluated the amount of mitochondria in total BAT homogenates. As reported in Figures 3B–D, we found lowered levels of mitochondrial proteins (Figure 3B), decreased citrate synthase activity (Figure 3C) and diminished mtDNA content (Figure 3D). Accordingly, reduced levels of the uncoupling protein UCP1 and mitochondrial biogenetic marker PGC-1α were observed (Figure 3B), suggesting that dietary fat overload during pregnancy and lactation induces a significant mitochondrial mass pauperization in BAT of pups. Furthermore, by NADH-diaphorase staining we confirmed the diminished mitochondrial quantity (Figure 3B). In HFD-f1 mice, the metabolic reorganization of mitochondria was characterized by a slight reduction in oxygen consumption (Figure 3E), whereas ATP levels were increased (Figure 3F), thus setting a high bioenergetics state of BAT (Figure 3E). Interestingly, HFD-f1 displayed increased expression of n(DNA)-encoded mitochondrial OxPHOS genes (Figure 4A) as well as augmented OxPHOS equipment (Figure 4B), oxidative efficiency (Figure 4C), and citrate synthase activity (Figure 4D) in crude mitochondria.

### HFD-offspring mice weaned in ND partially rescue mitochondrial abnormalities and display cold intolerance

**Figure 5:**
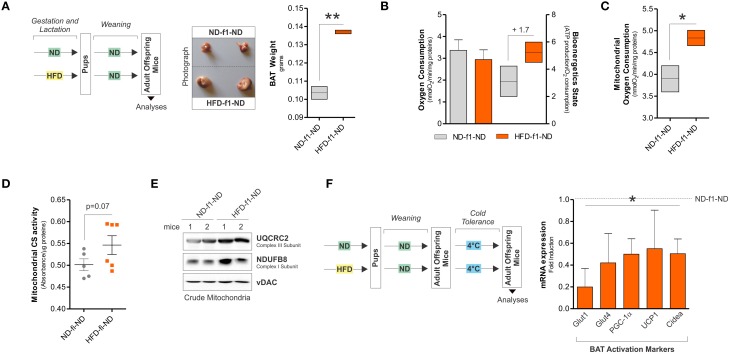
**Adult offspring mice weaned with ND partially recover mitochondrial abnormalities and display impaired thermogenic program**. **(A)** Schematic model of dietary approach in female adult offspring CD1 mice (left) and photographs of BAT aspect (center) and weight (right). **(B)** Real time oxygen consumption measured by a polarographic method and bioenergetics state expressed as ATP content/O_2_ consumption in total BAT lysates. **(C)** Real time mitochondrial oxygen consumption measured by a polarographic method in BAT crude mitochondria. **(D)** Citrate synthase (CS) activity measured by spectrophotometer in BAT crude mitochondria. **(E)** UQCRC2 and NDUFB8 analyzed by Western blot in BAT crude mitochondria. **(F)** Schematic model of cold experiment in adult offspring CD1 mice (left) and mRNA expression of BAT activation markers (right). vDAC served as loading control. Bar graphs are expressed as mean ±S.D. (*n* = 3 mice per group; ^*^p < 0.05; ^**^p < 0.01 vs. ND-f1-ND) ND-f1-ND, normal diet offspring weaned with ND; HFD-f1-ND, high fat diet offspring weaned with normal diet.

We then moved at evaluating the mitochondrial rearrangements of BAT after weaning with normal diet (HFD-f1-ND). HFD-f1-ND maintained different body weight (14.5 ± 0.6 vs. 12.6 ± 3.2 g), enlarged BAT mass (Figure 5A) and the reduction of mitochondrial amount (data not shown) with respect to ND-f1-ND mice. Furthermore, weaning with ND restored the rate of oxygen consumption even though the bioenergetics state remained increased (Figure 5B). Moreover, oxygen consumption measured on crude mitochondria of HFD-f1-ND mice was higher than ND-f1-ND (Figure 5C). Additionally, no variations in citrate synthase activity (Figure 5D) and an increase of OxPHOS proteins were detected in HFD-f1-ND (Figure 5E). Finally, to test the efficiency of thermogenic program ignition in BAT, we exposed HFD-f1-ND offspring mice to cool temperature (4°C). As reported in Figure 5F, thermogenic hallmarks such as PGC-1α, UCP1, Cidea, Glut1, and Glut4 were markedly down-regulated in cold-exposed HFD-f1-ND mice, suggesting that chronic dietary fat excess imposes BAT unresponsiveness.

### HFD modifies mitochondrial network through nuclear FoxO1 modulation

**Figure 6:**
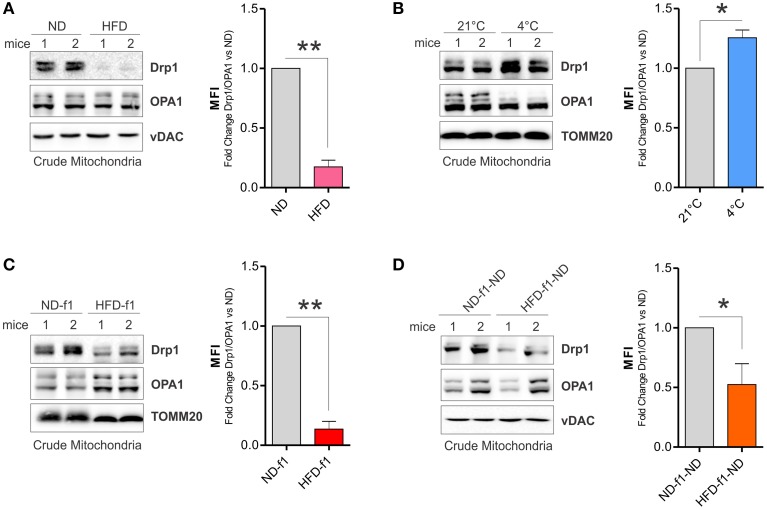
**HFD affects mitochondrial network in adult and offspring mice. (A,B)** Drp1 and OPA1 proteins analyzed by Western blot in BAT crude mitochondria after HFD **(A)** or cold exposure **(B)** in adult mice and relative calculation of MFI (right). **(C)** Drp1 and OPA1 proteins analyzed by Western blot in BAT crude mitochondria of pups grown in HFD and relative calculation of MFI (right). **(D)** Drp1 and OPA1 proteins analyzed by Western blot in BAT crude mitochondria of adult offspring mice weaned with ND and relative calculation of of MFI (right). TOMM20 and vDAC served as loading controls. Bar graphs are expressed as mean ±S.D. (*n* = 3 mice per group; ^*^p < 0.05 and ^**^p < 0.01 vs. controls). HFD, high fat diet; ND, normal diet. HFD-f1, high fat diet offspring; ND-f1, normal diet offspring; HFD-f1-ND, high fat diet offspring weaned with normal diet; ND-f1-ND, normal diet offspring weaned with normal diet; MFI, Mitochondrial Fragmentation Index (stoichiometric ratio between Drp1 and OPA1).

Mitochondrial fragmentation in brown adipose cells precedes mitochondrial uncoupling induced by adrenergic stimulation. Drp1 is an essential GTP-ase involved in mitochondrial fission (Otera and Mihara, 2011). It has been demonstrated that Drp1 plays a leading role in the control of mitochondrial fragmentation and uncoupling activity in brown adipocyte mitochondria (Gao and Houtkooper, 2014; Wikstrom et al., 2014). Indeed, uncoupled respiration and energy dissipation is impaired when a dominant-negative form of Drp1 is expressed in brown adipocytes (Gao and Houtkooper, 2014). Herein, we demonstrated that HFD strongly reduced Drp1 levels, whereas mitochondrial pro-fusion protein OPA1 was unaffected in BAT-crude mitochondria of adult mice (Figure 6A). By checking other fission and fusion markers (i.e., Fis1 and Mfn1) we did not detect any significant changes (data not shown). On the basis of these findings, we expressed mitochondrial reorganization as a Mitochondrial Fragmentation Index (MFI). In particular, we calculated MFI as ratio between Drp1 and total OPA1 isoforms levels in crude mitochondria. We calculated MFI in BAT mitochondria of HFD-treated mice and, as showed in Figure 6A right, a significant drop of MFI was achieved. To validate this index, we analyzed Drp1 and OPA1 protein levels after cold exposure in BAT mitochondria, which notably undergo fragmentation to mediate thermogenesis program. As expected, cool temperature increased Drp1 and diminished total OPA1 isoforms levels in BAT mitochondria, thus a significant MFI increase was registered (Figure 6B). These results pinpoint different mitochondria dynamics of BAT after dietary fat overload and highlight MFI as an effective tool to analyze mitochondrial dynamic. Similarly to adult mice, mitochondria from HFD-f1 also harbored reduced Drp1 level (Figure 6C) thus having a lower MFI with respect to ND-f1 (Figure 6C, right). Finally, we evaluated the impact of weaning with ND on mitochondrial Drp1 levels and MFI. Notably, although lesser markedly than HFD-f1 mice (Figure 6C), also mitochondria of HFD-f1-ND mice were characterized by a reduction of MFI consequential to diminished Drp1 protein levels (Figure 6D). These data suggest that dietary fat excess strongly impacts on mitochondrial dynamics mainly affecting Drp1 levels in crude mitochondrial fractions.

**Figure 7:**
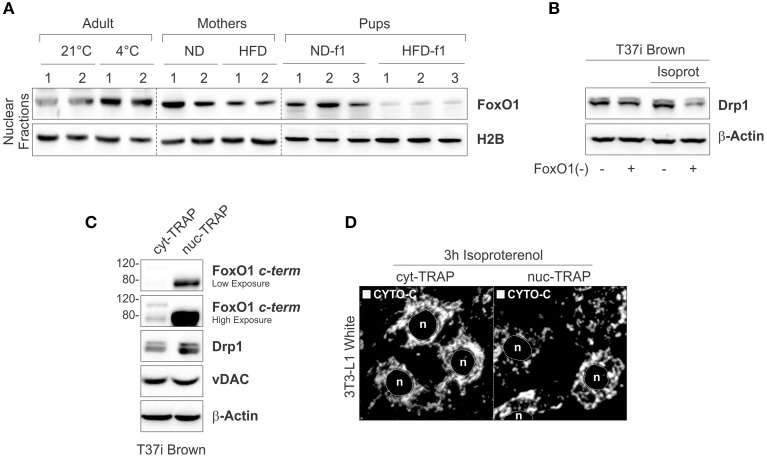
**Nuclear FoxO1 governs mitochondrial drp1 in brown fat cells. (A)** FoxO1 protein analyzed by Western blot in nuclear fractions of BAT. **(B)** Drp1 protein analyzed by Western blot in total lysates of scramble or FoxO1(–) T37i brown adipocytes treated with isoproterenol. **(C)** FoxO1, Drp1, and vDAC proteins analyzed by Western blot in total lysates of ^cyt−TRAP^FoxO1 or ^nuc−TRAP^FoxO1 T37i brown adipocytes. **(D)** Mitochondrial morphology analyzed by confocal microscopy in ^cyt−TRAP^FoxO1 or ^nuc−TRAP^FoxO1 3T3-L1 white adipocytes treated with isoproterenol and stained using cytochrome c (CYTO-C) antibody. *In vitro* experiments were performed at least four time and representative images are reported. β-Actin and H2B served as loading controls. HFD-f1-ND, high fat diet offspring weaned with normal diet; ND-f1, normal diet offspring weaned with normal diet.

Nuclear FoxO1 redistribution governs several metabolic routes in adipose tissue including the induction of the thermogenic program in BAT (Nakae et al., 2008; Ortega-Molina et al., 2012). In our work we compared the levels of nuclear FoxO1 (^n^FoxO1) in BAT upon HFD or cold exposure. As reported in Figure 7A, cold caused ^n^FoxO1 accumulation, whereas HFD induced a reduction of ^n^FoxO1 levels in mothers as well as in HDF-f1 pups (Figure 7A). In order to link FoxO1 to mitochondrial dynamic in BAT, we analyzed Drp1 in T37i brown adipocytes downregulating FoxO1 [T37i^FoxO1(−)^] and treated with the adrenergic agonist isoproterenol (ISO). In T37i^FoxO1(−)^, mitochondrial Drp1 was reduced upon ISO treatment (Figure 7B), implying FoxO1 in the control of Drp1 levels. In line with the obtained data, T37i overexpressing a constitutively active nuclear form of FoxO1 (FoxO1^nuc−TRAP^) showed higher Drp1 levels than T37i carrying cytoplasm-trapped mutant of FoxO1 (FoxO1^cyt−TRAP^; Figure 7C). Accordingly, ISO-treated 3T3-L1 white adipocytes overexpressing the FoxO1^cyt−TRAP^ displayed a more fused mitochondria network than adipocytes carrying the FoxO1^nuc−TRAP^ (Figure 7D). These results highlight a potential key role of FoxO1 in governing mitochondrial dynamics during BAT activation.

## Discussion

Dietary patterns during pregnancy significantly impact on fetal and offspring growth (Desai and Ross, 2011; Tzanetakou et al., 2011). In the offspring maternal HFD increases the risk of developing obesity and metabolic syndrome later in life, thereby contributing to the reducing in life expectancy (Attig et al., 2013). Furthermore, also nutrition in early stage of life, such as lactation, affects later child health outcomes including obesity and T2D (Jiang et al., 2013; Kagohashi and Otani, 2015). However, the BAT metabolic rearrangements that link maternal HFD with age-related disorders are neglected.

In our work we initially revealed that in adult mice dietary fat overload affects mitochondrial oxygen consumption in BAT. Importantly, this was not due to changes in the abundance of mitochondria per amount of tissue but, since there is much more tissue, it can be assumed that HFD-fed mice rather have an increased total mitochondrial mass in BAT. In accordance with reduced oxygen consumption, mitochondria of adult mice treated with HFD also showed a weakened proton gradient production. Generally, mitochondrial oxygen consumption is strongly related to electron transport flux and proton gradient formation with UCP1 exerting depolarization and thermogenic activity (Bartesaghi et al., 2015). Mitochondria of adult mice treated with HFD showed a membrane depolarization that is independent of UCP1 activation. Such phenomenon was mainly consequential to the reduction of OxPHOS proteins within mitochondria likely owing to reduced gene transcription of both mt(DNA)- and n(DNA)-encoded OxPHOS genes. A potential explanation for the observed lower respiration rate of mitochondrial BAT exposed to fat overload is that it might serve as an adaptation mechanism for avoiding excessive reactive oxygen species (ROS) production (Adjeitey et al., 2013). Indeed, nutrient or calorie excess has deleterious effect on mitochondrial integrity impinging electron transport along OxPHOS complexes inevitably leading to increase in ROS production and damage to mitochondria (Hu and Liu, 2011; Tiganis, 2011).

The analysis of mitochondrial responses observed in pups exposed to HFD, during pregnancy and lactation, revealed a diverse mitochondrial rearrangement both in terms of mass as well as in functionality when compared to mothers. Indeed, pups mice displayed reduced mitochondrial content and higher oxidative capacity of BAT mitochondria, suggesting that dietary fat excess differently impacts on mitochondrial responses in relation to the developmental process. Actually, fatty acids could modulate epigenetic processes during critical ontogenic periods leading to alteration of the transcription of genes that represent tissue-specific marks (Burdge and Lillycrop, 2014). Accordingly, HFD resulted in alterations in substrate utilization in the mothers, whereas prolonged exposure to HFD resulted in changes of structure as well as in gene expression within metabolically important organs in the offspring (Williams et al., 2014). However, we cannot exclude that during gestation other maternal factors such as hyperglycemia and hyperinsulinemia may contribute independently of fatty acids overload to the BAT phenotype and dysfunction observed in the offspring. Interestingly, we recovered the bioenergetics state in BAT after HFD exposure. This metabolic adaptation could be functionally associated with increased energy demand in BAT when chronically exposed to fat overload (Liesa and Shirihai, 2013). In particular, we suggest that the higher bioenergetics state of BAT might be associated with the induction of biosynthetic processes to dampen fat toxicity.

Notably, weaning with ND was marginally efficient in reverting the mitochondrial responses. In particular, adult offspring mice grown under dietary fat excess, only partially recovered mitochondrial functionality if weaned with ND. However, even if weaned with ND, mice exposed to HFD during gestation and lactation exhibited an inactivation of thermogenic program after cold exposure. This result could be explained by an unrecovered mitochondrial dynamic, which was characterized by higher MFI. Such mitochondrial adaptations could suggest the existence of a potential epigenetic reprogramming in pups exposed to fat excess during the early stage of life, which are maintained in adult age (Lillycrop and Burdge, 2012).

Mitochondrial bioenergetics has been shown to cause variations in mitochondrial dynamics (Liesa and Shirihai, 2013). A lower bioenergetics state induced by β_3_-receptor agonist (i.e., norepinephrine) triggers Drp1-mediated mitochondrial fragmentation in brown adipocytes (Wikstrom et al., 2014). Remarkably, overexpression of a dominant negative form of Drp1 (inactivation of its GTPase domain) decreased mitochondrial fragmentation and oxygen consumption in brown fat cells, highlighting Drp1 as a key mediator of mitochondrial reorganization and energy expenditure in BAT. In line with these data, we observed higher Drp1 protein levels in mitochondria of BAT exposed to cold. Intriguingly, dietary fat excess, which increased bioenergetics state of BAT, was associated with diminished mitochondrial protein levels of Drp1. These results suggest that the reorganization of mitochondrial network could be an adaptive response to restore the oxidative efficiency of mitochondria chronically exposed to fat excess. Furthermore, we proposed a Mitochondrial Fragmentation Index (MFI) to briefly and simply determine a mitochondrial dynamic in an *in vivo* system in which mitochondrial morphology analysis requires more complex techniques (e.g., TEM). Interestingly, dietary fat excess caused significant reduction of MFI in mothers as well as in offspring mice, suggesting the occurrence of a potential mitochondrial fusion in BAT. In some cell types mitochondrial fusion generates large, extended mitochondrial networks that constitute electrically coupled systems (Westermann, 2012). Mitochondrial fusion allows efficient mixing of mitochondrial content, and represents an advantageous rearrangement during high-energy demand (Westermann, 2012). Indeed, this mitochondrial reorganization was proposed to operate as intracellular power-transmitting cables. It was shown that some environmental stressors could trigger increased mitochondrial fusion, a process termed stress-induced mitochondrial hyperfusion. This process is accompanied by augmented ATP production, suggesting that fusion is necessary to optimize mitochondrial efficiency in order to cope increased energy demand during stress conditions (Westermann, 2012).

The transcription factor FoxO1 quickly and dynamically responds to external stimuli. Nutrient shortage promotes FoxO1 redistribution into nuclei thus enhancing its transcriptional capacity (Lettieri Barbato et al., 2013, 2014a,b). Differently, high insulin levels mediate FoxO1 nuclear exclusion through its phosphorylation by Akt (Zhao et al., 2004). In brown adipocytes, forced FoxO1 nuclear accumulation up-regulates markers of thermogenesis activation (Ortega-Molina et al., 2012). Oppositely to cold exposure, we show that chronic dietary fat overload reduces ^n^FoxO1 levels in brown adipose cells along with diminished Drp1 protein. These data suggest that under calorie excess, FoxO1 could translocate from nucleus to cytoplasm participating in mitochondrial morphology rearrangement. A potential mechanism could be ascribed to high insulin levels induced by HFD. Indeed, by activating Akt signaling cascade insulin mediates both mitochondrial fusion and FoxO1 nuclear exclusion (Lettieri Barbato et al., 2014b).

**Figure 8:**
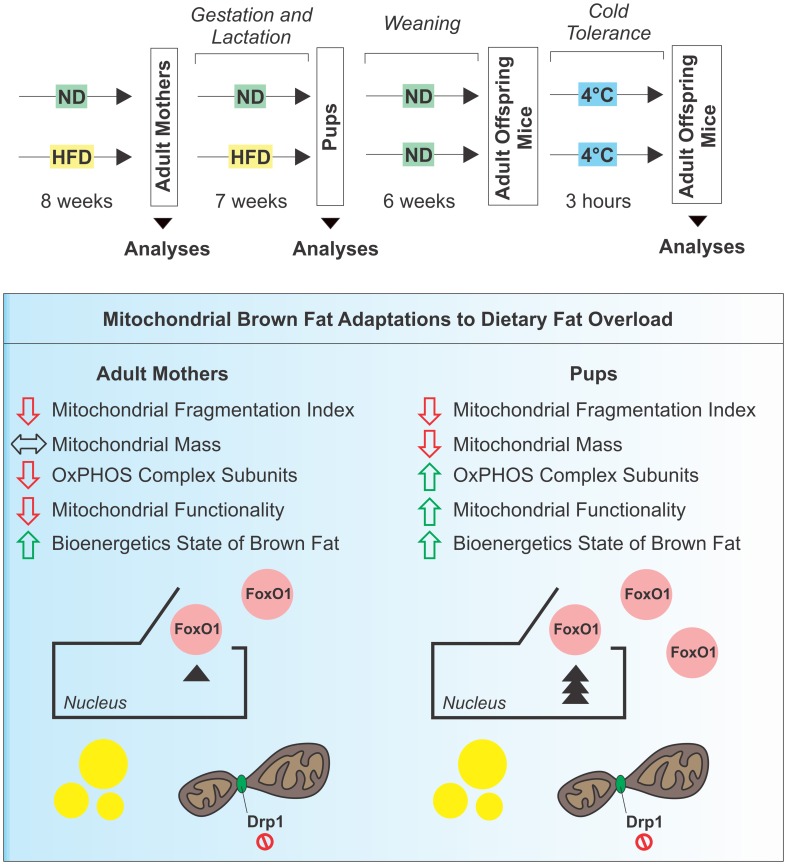
**Schematic representation of experimental design and mitochondrial BAT responses to dietary fat overload**.

Our findings demonstrate that BAT mitochondria have great metabolic plasticity as they efficiently and differently adapt to nutritional changes in dependence of the developmental stage (Figure 8). In the mothers, HFD does not influence mitochondrial mass but causes an impairment of mitochondrial functionality mainly due to pauperization of OxPHOS equipment. In pups exposed to HFD during gestation and lactation, although reduction of mitochondrial amount occurs in BAT, mitochondrial functionality is enhanced and directed to produce ATP rather than to support thermogenic program. However, in adult offspring mice the replacement with ND only partially revert the observed mitochondrial adaptations.

Our findings are of broad interest in the field of metabolic disorders and suggest that maternal high fat diet may have deleterious consequences on body metabolism in the offspring also by affecting BAT-mediated energy expenditure. Given the emerging anti-diabetic and anti-obesity role of BAT (Tseng et al., 2010), our results highlight BAT dysfunction as a possible contributing factor in increasing susceptibility in developing age-related metabolic disorders. Future studies are however still necessary to better understand the mechanisms by which modern dietary patterns impact on BAT functionality during early stages of life thus modulating health and lifespan.

## Author contributions

DL designed research and write the manuscript. GT and RV performed *in vivo* and *in vitro* experiments. SC and SB performed histochemical assay and analyzed the data. KA and MC supervised research, contribute to write the manuscript, analyzed the data, and provided intellectual input.

## Funding

This work was supported by grants from MIUR-PRIN (20125S38FA_002) and Ministero della Salute (GR-2011-02348047).

### Conflict of interest statement

The authors declare that the research was conducted in the absence of any commercial or financial relationships that could be construed as a potential conflict of interest.
